# A subtype of cancer‐associated fibroblast expressing syndecan‐2 (SDC2) predicts survival and immune checkpoint inhibitor response in gastric cancer

**DOI:** 10.1002/ctm2.70079

**Published:** 2024-12-13

**Authors:** Ji‐Yong Sung, Jae‐Ho Cheong, Kihye Shin, Eui Tae Kim

**Affiliations:** ^1^ Center for Genome Engineering, Institute for Basic Science (IBS) Yuseong‐gu Daejeon Republic of Korea; ^2^ Department of Surgery Yonsei University College of Medicine Seoul Republic of Korea; ^3^ Department of Microbiology and Immunology Jeju National University College of Medicine Jeju Republic of Korea; ^4^ Jeju Research Center for Natural Medicine, Jeju National University Core Research Institute Jeju Republic of Korea

1

Dear Editor,

We explored the heterogeneity and clinical implications of cancer‐associated fibroblasts (CAFs) within the gastric cancer microenvironment to understand their contributions to tumour progression and therapeutic resistance. CAFs, essential components of the tumour microenvironment, regulate tumour growth, immune responses, and therapeutic outcomes. We classified CAFs into three subtypes: myofibroblastic (myoCAF), immune‐regulatory (irCAF), and inflammatory (infCAF), based on gene expression signatures and functional characteristics (Figure 1A–C). Each CAF subtype was characterized by unique marker genes from a literature review (Tables S1 and S2).^1^ Gene ontology (GO) analysis revealed that transcription factors NFKB1, RELA, SP1, JUN, and transcriptional regulator HDAC2 are key in CAF subtype regulation (Figure 1B). Using the MCODE algorithm^2^ for protein–protein interaction, we found myoCAFs were involved in blood vessel development and extracellular matrix modelling, while irCAFs were associated with peptide ligand‐binding and chemokine receptors, indicating distinct roles in immune modulation (Figure 1D,E).

**FIGURE 1:**
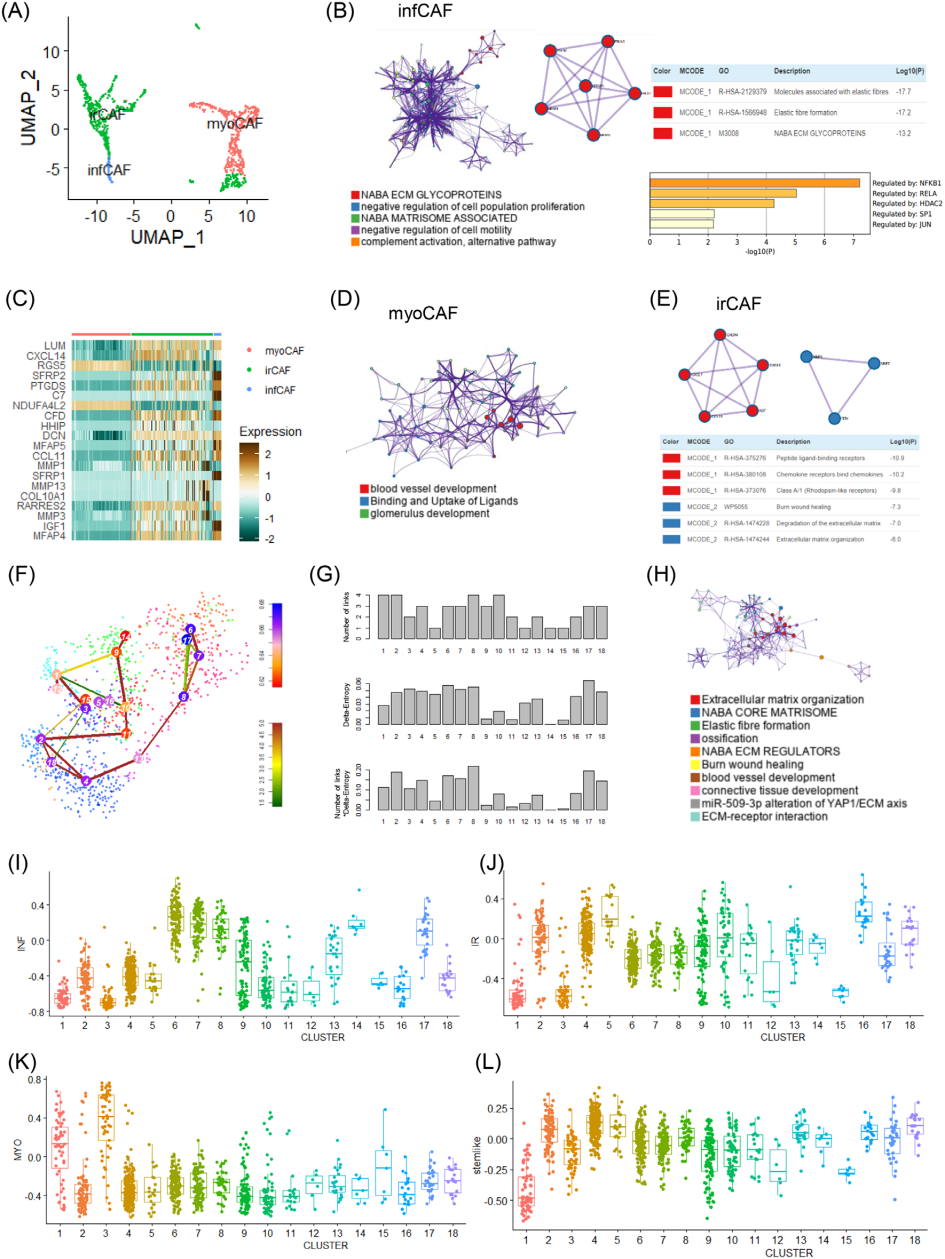
Distinct gene signatures of cancer‐associated fibroblast subtypes. (A) UMAP plot illustrating three CAF subtypes (irCAF, myoCAF, and infCAF) in cohort 1. (B) Gene ontology network for infCAF, accompanied by a table for protein–protein interactions and a bar graph for transcription factors. (C) Heatmap displaying marker genes of the three CAF subtypes (irCAF, myoCAF, and infCAF). (D) Gene ontology network for myoCAF. (E) MCODE analysis results show protein–protein interactions in irCAF. (F) t‐SNE plot depicting high entropy and high stemness activated fibroblasts in cohort 2. (G) Bar graph illustrating stemness clusters in cohort 2. (H) Gene ontology network for high‐stemness fibroblasts in cohort 2. (I) Box plot presenting inflammatory signature scores in fibroblast clusters of cohort 2. (J) Box plot presenting immune regulatory signature scores in fibroblast clusters of cohort 2. (K) Box plot presenting myofibroblastic signature scores in fibroblast clusters of cohort 2. (L) Box plot presenting stem‐like signature scores in fibroblast clusters of cohort 2.

To further investigate CAF stemness, we employed the StemID tool on single‐cell cohorts. Clusters 17 and 8 showed notably high entropy and stemness, indicating that these fibroblasts are highly activated and possess stem‐like properties, contributing to tumour progression and treatment resistance (Figure 1F,G). These fibroblasts were enriched in pathways related to extracellular matrix organization,^3^ including the NABA core matrisome and elastic fibre formation (Figure 1H).^4^ Our analysis further revealed a strong correlation between irCAF signatures and stem‐like signatures in bulk gastric cancer samples, suggesting that irCAFs play a key role in sustaining an aggressive tumour microenvironment (Figure 1I‐L).^5^

We evaluated the impact of these CAF subtypes on patient prognosis using data from the Cancer Genome Atlas (TCGA) stomach adenocarcinoma (STAD) dataset. High expression of infCAF, irCAF, and myoCAF signatures was linked to poor prognosis (Figure 2A). A combined analysis of all three CAF signatures also indicated poor outcomes for the high‐expression group (Figure 2B). In the Yonsei Cancer Hospital cohort of 497 gastric cancer patients (Y497 dataset), these CAF subtypes were enriched in the stem‐like type (Figure 2C). While the infCAF signature was not significantly linked to adverse outcomes, high expression of both irCAF and myoCAF signatures was consistently linked to worse clinical outcomes (irCAF: *p* = .0022, myoCAF: *p* = .0053) (Figure 2D). We classified patients into nine groups based on the CAF subtype signature via gene set enrichment test in the Y497 cohort. (Figure 2E). Furthermore, we confirmed that the CAF subtype signatures found in TCGA pan‐cancer datasets act differently across various cancer types. All three CAF signatures in BLCA, LGG, and STAD cancer types were significant for patient prognosis (Figure 2F). Using Y497 transcriptome profiling, we performed a deconvolution analysis to understand how different CAF subtypes contribute to the immune tumour microenvironment. Both infCAF and irCAF showed similar trends in stem‐like type samples, being positively correlated with macrophage M2 and regulatory T (Treg) cells, while CD8+ T cells were more prevalent in the gastric molecular subtype (Figure 2G).

**FIGURE 2:**
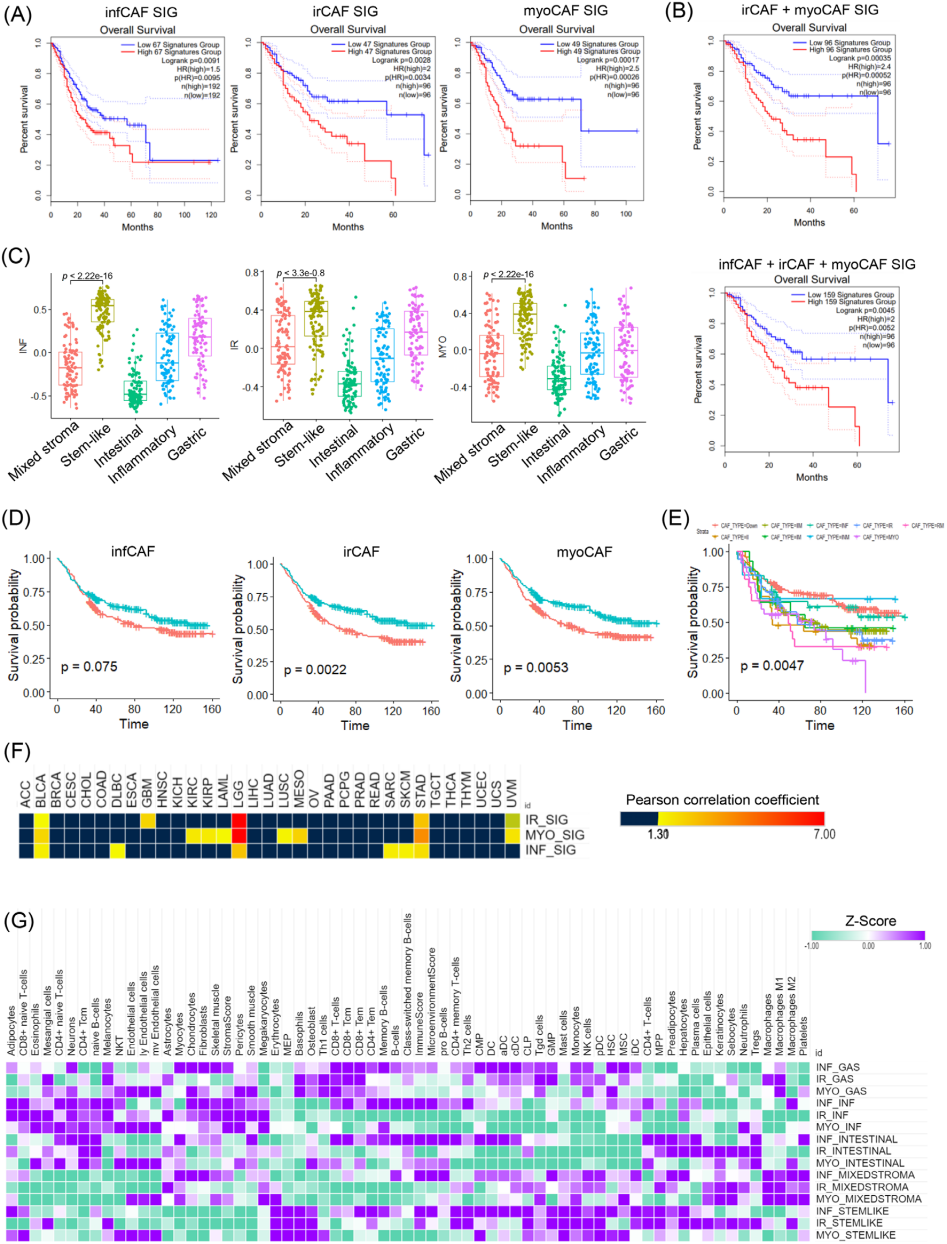
Clinical relevance of CAF signatures. (A) Kaplan–Meier survival plots showing the overall survival rates of patients classified by CAF signatures (infCAF, irCAF, and myoCAF) in the TCGA STAD dataset. The p‐value was calculated using the log‐rank test. (B) Kaplan–Meier survival plots for combined CAF signatures in the TCGA STAD dataset. (C) Box plot illustrating the distribution of each CAF signature across five molecular subtypes (mixed stroma, stem‐like, intestinal, inflammatory, and gastric). (D) Kaplan–Meier survival plots each CAF signature in the 97 cohort, with groups divided into low and high expression (infCAF: Low 260 samples, High 237 samples, irCAF: Low 256 samples, High 241 samples, myoCAF: Low 252 samples, High 245 samples). (E) Kaplan–Meier survival plots for nine CAF subtypes in the Y497 cohort (Down: 195 samples; II: 25 samples; IIM: 140 samples; IM: 14 samples; INF: 35 samples; INM: 6 samples; IR: 35 samples; MYO: 27 samples; RM: 20 samples). (F) Heatmap of *p*‐value from survival analysis in TCGA pancreatic cancer dataset. (G) Heatmap from tumour microenvironment deconvolution analysis, showing the relationship between 3 CAF subtypes in five molecular subtypes.

To understand the influence of CAFs on immune response and therapy resistance, we conducted single‐cell analysis on non‐responders to immune checkpoint blockade (ICB) therapy. High‐entropy, stem‐like activated cells were predominantly in clusters 1 and 7 (Figure 3A,B). Cluster 1 had mostly endothelial cells, while cluster 7 included fibroblasts, T cells, macrophages, and others (Figure 3C). Clusters 8 and 10, with the lowest stemness and entropy, were largely B cells linked to adaptive immunity. GO analysis of highly expressed genes within endothelial cells showed enrichment in pathways related to blood vessel development, cell migration, and VEGFA‐VEGFA2 signalling (Figure 3D). The prognostic relevance of these genes was confirmed in the TCGA STAD dataset, where high expression correlated with poor outcomes (Figure 3E). Additionally, these genes were enriched in the stem‐like type within the Yonsei Cancer Hospital cohort, demonstrating a consistent pattern (Figure 3F). We further assessed the expression of signature genes linked to drug resistance using bulk RNA‐seq data from non‐responder and responder groups to ICB treatment. The signature genes were mainly overexpressed in nonresponders (Figure 3G). The correlation between stem‐like signatures and CAF subtypes was validated using a gastric cancer patient‐derived organoid (PDO) dataset from Yonsei Cancer Hospital, showing that the immune‐regulatory signature had the strongest correlation with the stem‐like phenotype (Figure 3H,I). At the single‐cell level, irCAFs demonstrated the highest expression of stem‐like PDO signature genes, indicating their potential role in drug resistance (Figure 3J).

**FIGURE 3:**
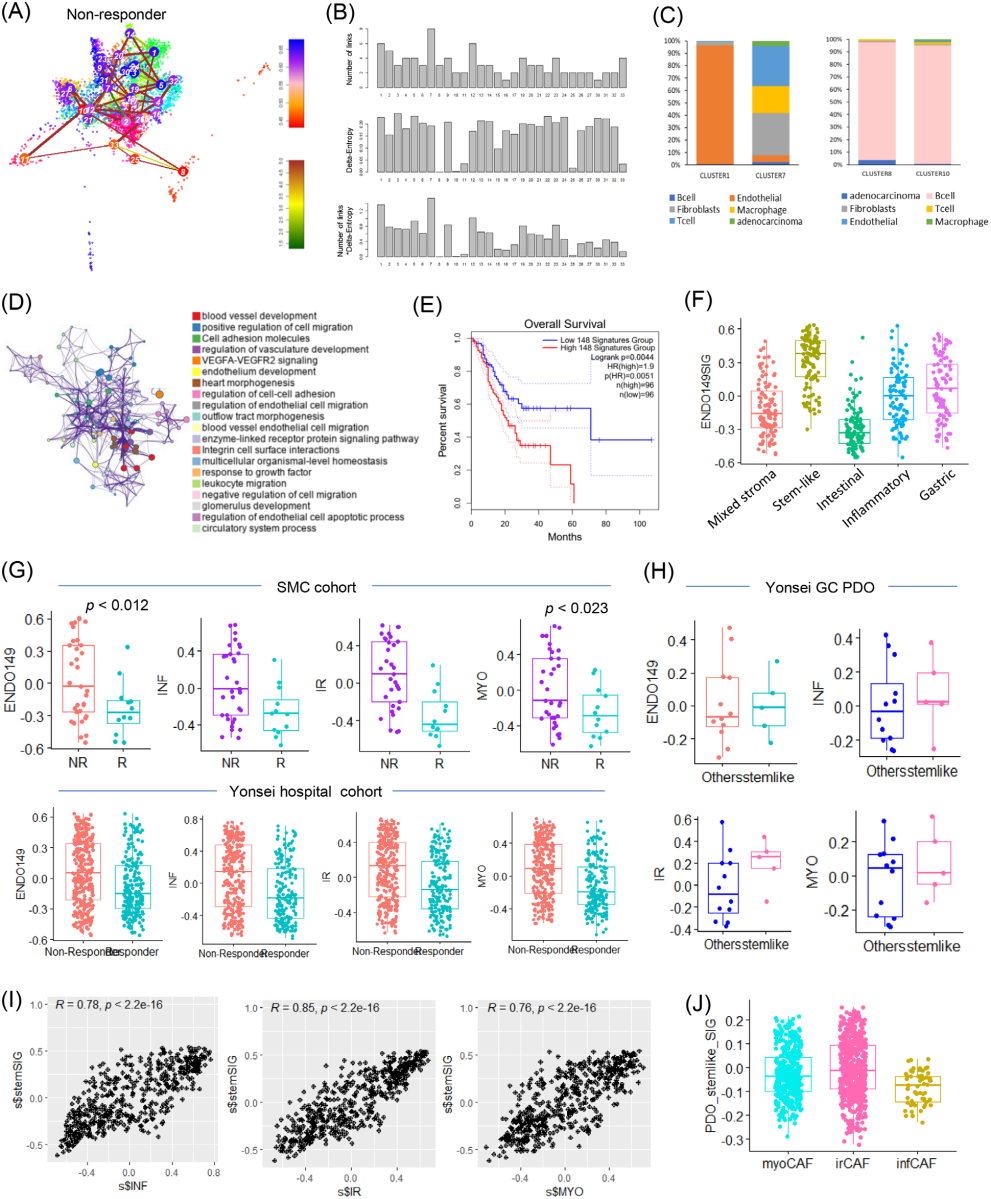
Activated high‐stemness cells enriched in endothelial cells and fibroblasts in non‐responders to immunotherapy at the single cell level. (A) t‐SNE plot for high entropy and stemness cluster (blue: high stemness, red: low stemness) for non‐responders. (B) Bar graph displaying the distribution of stemness clusters. (C) Bar graph showing the cell type fraction in the high stemness cluster (left) and the low stemness cluster (right). (D) Gene ontology network in highly enriched stem‐like cells in non‐responder group. (E) Kaplan–Meier survival plots for high entropy endothelial signatures in TCGA STAD dataset. (F) Box plot of 149 endothelial signature scores across 5 molecular subtypes. (G) Box plot comparing endothelial signature and CAF subtype signature between non‐responders and responders in the Samsung Medical Center (SMC) dataset and the Yonsei Cancer Hospital cohort. (H) Box plot comparing endothelial signature and CAF subtype signatures between other and stem‐like types in the Yonsei Cancer Hospital gastric cancer patient‐derived organoid (PDO). (I) Scatter plot showing the correlation between each CAF signature and stem‐like signature. (J) Box plot of stem‐like PDO signatures in CAF subtypes.

To decipher the communication patterns of CAFs within the tumour microenvironment, we employed Cellchat^6^ analysis to identify key signalling pathways (Figure 4A). We identified eight cell types that clustered into three main communication patterns, predicting the involvement of fibroblast‐derived ligands such as VEGF, PTN, and ANGPT (Figure 4B,C). Fibroblasts, as crucial components of the PTN signalling network, acted as both senders and receivers, particularly influencing mesenchymal stem cells, proliferative cells, peritoneal mesothelial cells, and tumour cells (Figure 4D). Within the CAF subtypes, PTN signalling was mediated by both irCAF and infCAF (Figure 4E). Predicted analysis of ligand‐receptor pairs identified the PTN ligand and syndecan‐2 (SDC2) receptor as critical components of the signalling pathway (Figure 4F). SDC2, a transmembrane proteoglycan involved in glycosaminoglycan metabolism, was found to be highly expressed in myoCAFs and cancer stem cells, correlating with poor prognosis in both the TCGA STAD dataset and the Y497 cohort

**FIGURE 4:**
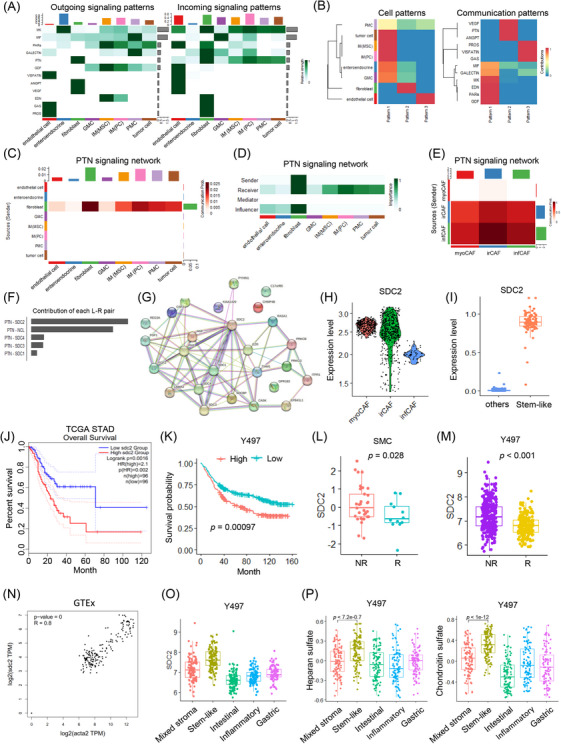
Intercellular communications for CAF subtypes link to PTN‐SDC2 signalling in stem‐like gastric cancer. (A) Heatmap illustrating outgoing and incoming signalling patterns in cohort 2. (B) Cell patterns and communication patterns identified in cohort 2. (C) Heatmap of the PTN signalling network in cohort 2. (D) PTN signalling pathway network showing the roles of sender, receiver, mediator, and influencer in cohort 2. (E) PTN signalling network specific to the three CAF subtypes in cohort 2. (F) Bar graph showing the contribution of each ligand–receptor pair within CAF subtypes. (G) Network diagram depicting protein–protein interactions involving *SDC2*. (H) Box plot showing *SDC2* expression levels across CAF subtypes. (I) Box plot comparing *SDC2* expression between other subtypes and stem‐like subtypes. (J) Kaplan‐Meier survival plots for *SDC2* expression in the TCGA STAD dataset. (K) Kaplan–Meier survival plots for *SDC2* expression in the Yonsei Cancer Hospital cohort. (L) Box plot comparing *SDC2* expression levels between non‐responders and responders in the SMC dataset. (M) Box plot comparing *SDC2* expression levels between non‐responders and responders in the Y497 cohort. (N) Correlation plot between *SDC2* and *ACTA2* expression in gastric cancer from the GTex dataset. (O) Box plot for *SDC2* expression levels across five molecular subtypes in the Yonsei Cancer Hospital cohort. (P) Box plot showing metabolic pathway activity across five molecular subtypes in the Yonsei Cancer Hospital cohort.

(Figure 4H–K). Elevated *SDC2* expression was particularly noted in ICB non‐responder groups within both the Samsung Medical Center (SMC) and Y497 cohorts^7^ (Figure 4L,M). A strong correlation (*R* = .8, *p* = 0) was observed between *SDC2* and the *ACTA2* gene, a marker associated with resistance to ICB therapy (Figure 4N).^8^ Furthermore, *SDC2* expression was notably enriched in the stem‐like type of gastric cancer, potentially promoting tumorigenesis via glycosaminoglycan biosynthesis pathways such as heparan sulfate and chondroitin sulfate (Figure 4O,P).^9^ The transition from anti‐tumorigenic syndecans to tumorigenic SDC2 may influence the invasive capacity and metastatic potential of highly aggressive tumour cells.^10^

In conclusion, our study highlights the heterogeneity of CAF subtypes in gastric cancer and their roles in prognosis and therapy resistance. Targeting CAFs could provide new avenues for personalised treatment of gastric cancer.

## AUTHOR CONTRIBUTIONS

JYS was responsible for the conceptualisation, methodology development, data analysis, manuscript drafting, wrote, review, editing, interpretation of results and supervision of the study. JHC contributed to the generation of PDO data and manuscript review and contributed to the interpretation of results. KS performed contributed to manuscript review.ETK provided funding acquisition, manuscript review and editing, and contributed to the interpretation of results.

## CONFLICT OF INTEREST STATEMENT

The authors declare no conflict of interest.

## ETHICS APPROVAL AND CONSENT TO PARTICIPATE

This study was approved by the Institutional Review Board of Yonsei Cancer Hospital (IRB no. 4‐2017‐0106). Informed consent was obtained from all participants.

## Supporting information



Supporting information

Supporting information

Supporting information

## Data Availability

The datasets generated and/or analyzed during this study are not publicly available. Researchers interested in the organoid RNA‐seq data for gastric cancer may contact Dr. Ji‐Yong Sung (5rangepineapple@gmail.com).
