# Supplementary material for: A subtype of cancer‐associated fibroblast expressing syndecan‐2 (SDC2) predicts survival and immune checkpoint inhibitor response in gastric cancer
Source: Clin Transl Med. 2024 Dec 13;14(12):e70079. doi: 10.1002/ctm2.70079 (PMC11645442; doi:10.1002/ctm2.70079)
Supplement: Supplementary file 1 — Supporting information [file CTM2-14-e70079-s001.docx]

**METHODS**

**Single cell RNA-seq analysis**

Two independent gastric cancer single-cell cohorts were utilized in this study. CAF subtypes were initially classified in the first cohort [1] and subsequently validated in the second cohort [2]. The "Seurat" R package was employed for data analysis, using its default parameter values [3]. Gene ontology analysis was conducted using Metascape [4]. To assess stemness at the single-cell level, the StemID tool was utilized [5]. Signature scores were calculated with the “GSVA” R package [6], and cell-cell interaction signaling pathways were identified using CellChat [7]. A comprehensive list of signature genes used for survival analysis is provided in Figures 2 and 3, as well as in the Supplementary Table. These genes were selected for their high expression levels in each CAF subtype, with a false discovery rate (FDR) of less than 0.001, determined using the Seurat package.

**Bulk RNA-seq analysis**

For the bulk RNA sequencing (RNA-seq) analysis, we utilized data from The Cancer Genome Atlas Stomach Adenocarcinoma (TCGA STAD) dataset and the Yonsei Cancer Hospital gastric cancer cohort, comprising 497 patients. Survival analysis was performed using the GEPIA2 platform [8] for TCGA data and the R package "survival" for the Yonsei Cancer Hospital gastric cancer cohort. Additionally, survival analysis was extended to the TCGA pan-cancer dataset using GEPIA2. Deconvolution analysis was conducted using the xCell tool (<https://comphealth.ucsf.edu/app/xcell>) [9] to infer the proportions of various cell types within the tumor microenvironment. ICB response result data were obtained from the Samsung Medical Center cohort [10] and the Y497 cohort (GSE 84437), with the ICB response of the Y497 cohort analyzed using TIDE [11]. The RNA-seq expression profiles of the Samsung Medical Center cohort (n=45) were normalized to fragments per kilobase of transcript per million (FPKM) and log10-transformed. The Y497 cohort, characterized by expression profiling by array, was classified into molecular subtypes based on specific marker genes: immune subtype (GZMB and WARS), epithelial subtype (CDX1), and stem-like subtype (SFRP4). Clinical outcomes varied among these subtypes, with the immune subtype showing the most favorable prognosis and resistance to chemotherapy, the epithelial subtype exhibiting intermediate prognosis and sensitivity to chemotherapy, and the stem-like subtype demonstrating the poorest prognosis and resistance to both standard chemotherapy and immune-directed therapy [12, 13]. Correlation analysis was performed using the R software (version 4.2.1). For RNA-seq analysis of gastric cancer organoids, we processed 17 samples, distinguishing them using our stem-like type classification method. The demultiplexed library reads were aligned to the human reference genome (GRCh38) using the STAR aligner program (version 2.7.3a) [14]. Gene expression quantification was conducted using the "GeneCount" function of the STAR software and htseq-count, with all genes annotated using the GRCh38 GTF format.

**Production and cultivation of normal and malignant gastric organoids**

Following approval from the Institutional Review Board (IRB No. 4-2017-0106), clinical samples for organoid development were obtained from patients at Yonsei Cancer Hospital who provided informed consent. Healthy and malignant tissue samples were collected through surgical resection, endoscopic biopsy, or ascites drainage. Organoids were generated and maintained with minor modifications to previously described methods [15]. Surgical specimens were throughly rinsed with PBS and minced into 3 mm^3^ fragments using surgical instruments. The tumor fragments underwent enzymatic digestion at 37 °C for 60 minutes with 1.5% collagenase, while normal tissue fragments were treated with 10 mM EDTA at room temperature for 10 minutes.

Prior to plating, collected epithelial cells were rinsed with PBS containing 10% fetal bovine serum (FBS) to inactivate digestive enzymes. For organoid culture, ascites samples were centrifuged and rinsed three times with ice-cold PBS, and the sedimented cells were used. Gastric cells were encapsulated in Matrigel vesicles and covered with culture medium. The cultural conditions included Advanced Dulbecco’s Modified Eagle Medium/F12 medium (Invitrogen, Carlsbad, CA, USA), R-spondin-conditioned medium, and Wnt-conditioned medium, supplemented with gastric growth factors such as bone morphogenetic protein inhibitors, noggin (PeproTech, Cranbury, NJ, USA), GlutaMAX-I (Invitrogen), B27 (Invitrogen), TGF beta I A83-01 (TOCRIS, Minneapolis, MN, USA), ROCK inhibitors, nicotinamide (Sigma-Aldrich, St. Louis, MO, USA), N-acetylcysteine (Sigma-Aldrich), gastrin (Sigma-Aldrich), epidermal growth factor (PeproTech), and fibroblast growth factor 10 (R&D systems, Minneapolis, MN, USA). The media were replaced every three to four days. Subsequently, gastric organoids were passed every six to eight days, depending on their size. Organoid size was determined by averaging the shortest and longest diameters across the centers of the organoids [15].

**Reference**

1. Kim J, Park C, Kim KH, Kim EH, Kim H, Woo JK, Seong JK, Nam KT, Lee YC, Cho SY: **Single-cell analysis of gastric pre-cancerous and cancer lesions reveals cell lineage diversity and intratumoral heterogeneity**. *NPJ Precis Oncol* 2022, **6**(1):9.

2. Sathe A, Grimes SM, Lau BT, Chen J, Suarez C, Huang RJ, Poultsides G, Ji HP: **Single-Cell Genomic Characterization Reveals the Cellular Reprogramming of the Gastric Tumor Microenvironment**. *Clin Cancer Res* 2020, **26**(11):2640-2653.

3. Hao Y, Hao S, Andersen-Nissen E, Mauck WM, 3rd, Zheng S, Butler A, Lee MJ, Wilk AJ, Darby C, Zager M *et al*: **Integrated analysis of multimodal single-cell data**. *Cell* 2021, **184**(13):3573-3587 e3529.

4. Zhou Y, Zhou B, Pache L, Chang M, Khodabakhshi AH, Tanaseichuk O, Benner C, Chanda SK: **Metascape provides a biologist-oriented resource for the analysis of systems-level datasets**. *Nat Commun* 2019, **10**(1):1523.

5. Grun D, Muraro MJ, Boisset JC, Wiebrands K, Lyubimova A, Dharmadhikari G, van den Born M, van Es J, Jansen E, Clevers H *et al*: **De Novo Prediction of Stem Cell Identity using Single-Cell Transcriptome Data**. *Cell Stem Cell* 2016, **19**(2):266-277.

6. Hanzelmann S, Castelo R, Guinney J: **GSVA: gene set variation analysis for microarray and RNA-seq data**. *BMC Bioinformatics* 2013, **14**:7.

7. Jin S, Guerrero-Juarez CF, Zhang L, Chang I, Ramos R, Kuan CH, Myung P, Plikus MV, Nie Q: **Inference and analysis of cell-cell communication using CellChat**. *Nat Commun* 2021, **12**(1):1088.

8. Tang Z, Kang B, Li C, Chen T, Zhang Z: **GEPIA2: an enhanced web server for large-scale expression profiling and interactive analysis**. *Nucleic Acids Res* 2019, **47**(W1):W556-W560.

9. Aran D, Hu Z, Butte AJ: **xCell: digitally portraying the tissue cellular heterogeneity landscape**. *Genome Biol* 2017, **18**(1):220.

10. Kim ST, Cristescu R, Bass AJ, Kim KM, Odegaard JI, Kim K, Liu XQ, Sher X, Jung H, Lee M *et al*: **Comprehensive molecular characterization of clinical responses to PD-1 inhibition in metastatic gastric cancer**. *Nat Med* 2018, **24**(9):1449-1458.

11. Jiang P, Gu S, Pan D, Fu J, Sahu A, Hu X, Li Z, Traugh N, Bu X, Li B *et al*: **Signatures of T cell dysfunction and exclusion predict cancer immunotherapy response**. *Nat Med* 2018, **24**(10):1550-1558.

12. Cheong JH, Yang HK, Kim H, Kim WH, Kim YW, Kook MC, Park YK, Kim HH, Lee HS, Lee KH *et al*: **Predictive test for chemotherapy response in resectable gastric cancer: a multi-cohort, retrospective analysis**. *Lancet Oncol* 2018, **19**(5):629-638.

13. Sung JY, Cheong JH: **Gene signature related to cancer stem cells and fibroblasts of stem-like gastric cancer predicts immunotherapy response**. *Clin Transl Med* 2023, **13**(8):e1347.

14. Dobin A, Davis CA, Schlesinger F, Drenkow J, Zaleski C, Jha S, Batut P, Chaisson M, Gingeras TR: **STAR: ultrafast universal RNA-seq aligner**. *Bioinformatics* 2013, **29**(1):15-21.

15. Bartfeld S, Bayram T, van de Wetering M, Huch M, Begthel H, Kujala P, Vries R, Peters PJ, Clevers H: **In vitro expansion of human gastric epithelial stem cells and their responses to bacterial infection**. *Gastroenterology* 2015, **148**(1):126-136 e126.

**Declarations**

**Ethics approval and consent to participate**This study was approved by the Institutional Review Board (IRB No. 4-2017-0106) of Yonsei Cancer Hospital. Informed consent was obtained from all participants involved in the study.

**Availability of data and materials**
The datasets generated and/or analyzed during the current study are not publicly available. Researchers interested in accessing the organoid RNA-seq data for gastric cancer should contact Dr. Ji-Yong Sung ([5rangepineapple@gmail.com](mailto:5rangepineapple@gmail.com)).

**Competing interests**

The authors declare that they have no competing interests.

**Funding**

This research was supported by the National Research Foundation of Korea (NRF) under grant funded by the Korean Ministry of Science and ICT (RS-2024-00352590) and the Ministry of Education (RS-2023-00270936), Republic of Korea, and the Korea Health Technology R&D Project through the Korea Health Industry Development Institute (KHIDI) funded by the Ministry of Health & Welfare, Republic of Korea (RS-2022-KH129726, RS-2024-00438990).

**Authors' contributions**

JYS was responsible for the conceptualization, methodology development, data analysis, manuscript drafting, review, and editing as well as supervision of the study. KS performed data analysis and contributed to manuscript review and editing. JHC contributed to the generation of PDO data and manuscript review. ETK provided funding acquisition, manuscript review and editing, and contributed to the interpretation of results. All authors participated in the interpretation of the findings and approved the final manuscript.
